# Supplementary figures and images for: Multi-omics characterization of partial chemical reprogramming reveals evidence of cell rejuvenation
Source: eLife. 2024 Mar 22;12:RP90579. doi: 10.7554/eLife.90579 (PMC10959535; doi:10.7554/eLife.90579)

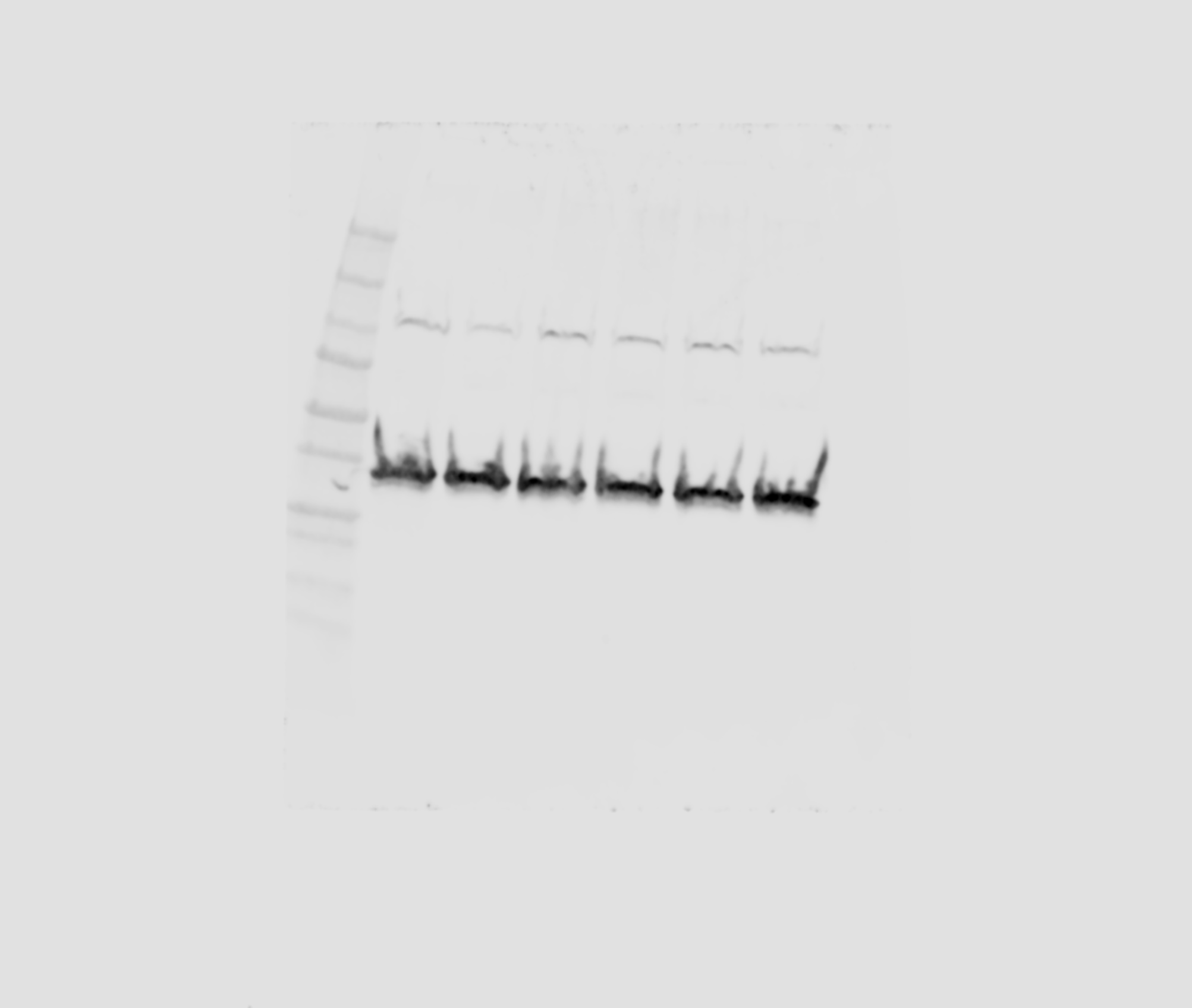

Supplement: Figure 5—source data 3. [file elife-90579-fig5-data3.zip › Figure 5 - source data 3/GADPH.tif]

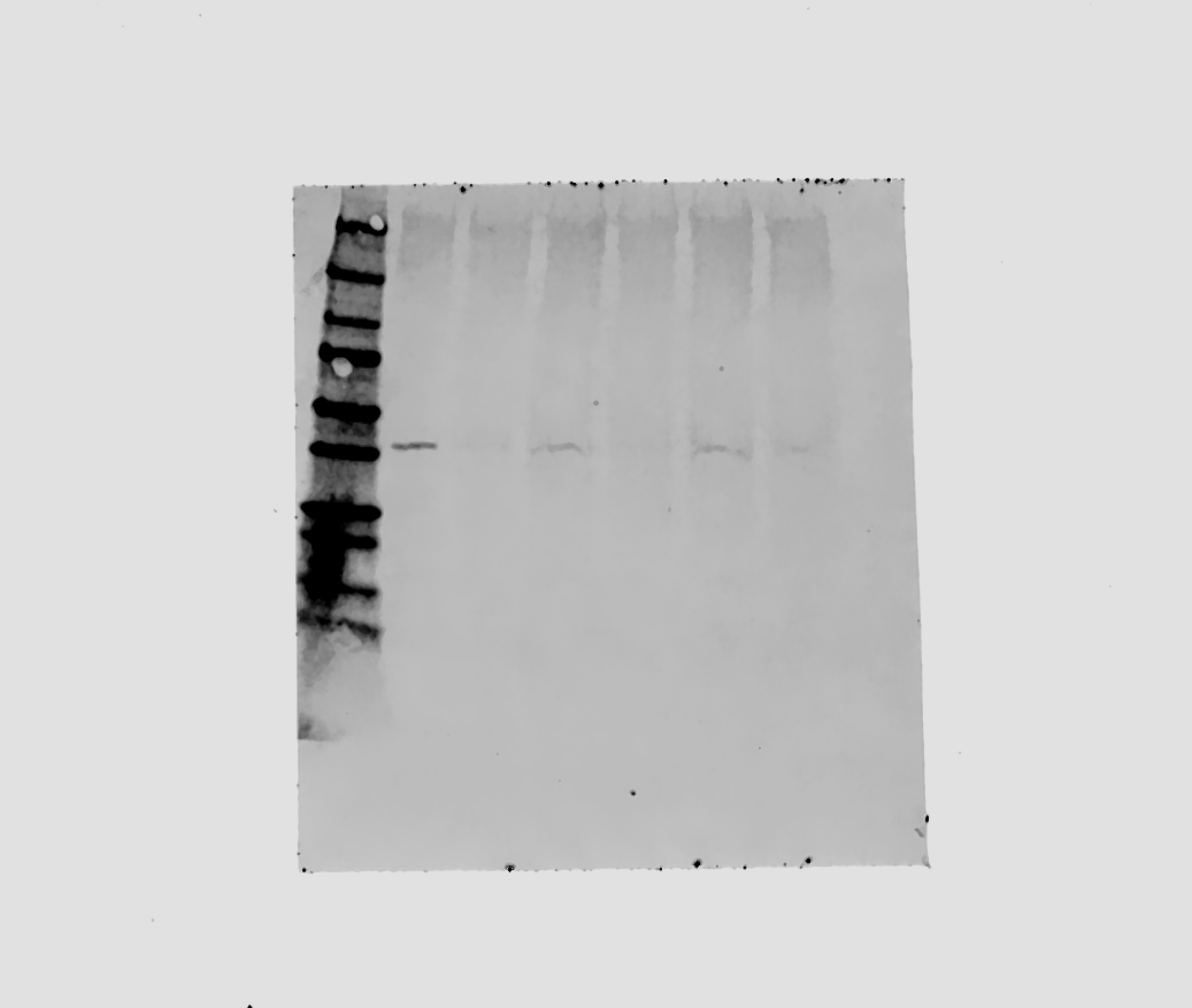

Supplement: Figure 5—source data 3. [file elife-90579-fig5-data3.zip › Figure 5 - source data 3/prkaca.tif]

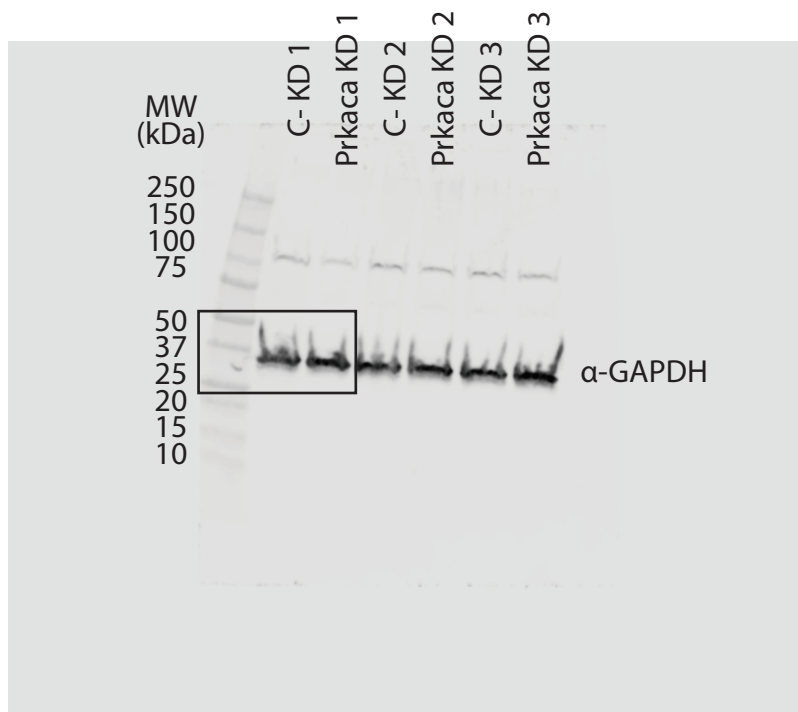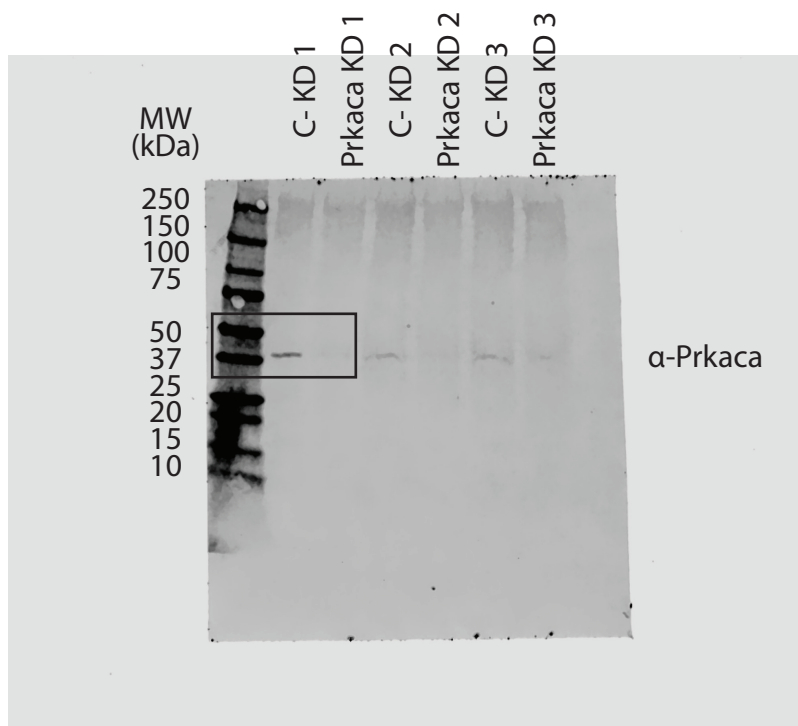

**B**

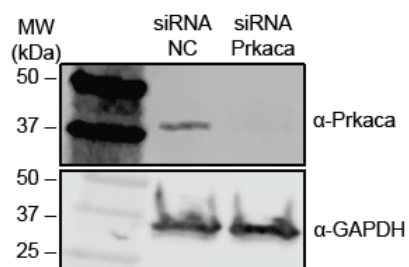

Supplement: Figure 5—source data 4. [file elife-90579-fig5-data4.zip › Figure 5 - source data 4/Figure 5 - source data 4.pdf]
